# Case 5/2019 - Naturally Evolving Ebstein’s Anomaly of Discrete Repercussion in 24-Year-old Asymptomatic Adult

**DOI:** 10.5935/abc.20190158

**Published:** 2019-09

**Authors:** Edmar Atik, Maria Angélica Binotto, Alessandra Costa Barreto

**Affiliations:** Instituto do Coração do Hospital das Clínicas da Faculdade de Medicina da Universidade de São Paulo, São Paulo, SP - Brazil

**Keywords:** Ebstein Anomaly, Tachycardia,Paroxysmal, Cardiac Output,Low, Tricuspide Valve Insufficiency, Echocardiography/methods, Magnetic Resonance Spectroscopy/methods

## Clinical data

Patient reports 3 episodes of paroxysmal tachycardia of up to 20 minutes, accompanied by precordial pain in the past 7 years, all with spontaneous reversal. Four years prior, the patient presented another tachycardia with signs of low output, including pallor, visual turbidity, cold extremities and mental confusion, requiring electrical reversion. On that occasion, ventricular tachycardia ablation in the right ventricular (RV) inflow tract through the right posterior accessory pathway was successfully performed. Ebstein’s anomaly diagnosis was given for the first time by echocardiography scan, at that time. Ever since, the patient remained asymptomatic, living a normal life and with no medication.

**Physical examination:** Good general condition, eupneic, acyanotic, normal pulses in the 4 limbs. Weight: 80 kg, Height: 180 cm, BP: 110 x 70 mmHg, HR: 70 bpm, Sat O_2_ = 94%.

**Precordium:** Apical impulse not palpated, with no systolic impulses. Normal heart sounds, with irregular splitting of the second heart sound. There were discrete systolic vibrations in the lower left sternal border. Unpalpable liver and clean lungs.

### Complementary tests

**Figure 1:**
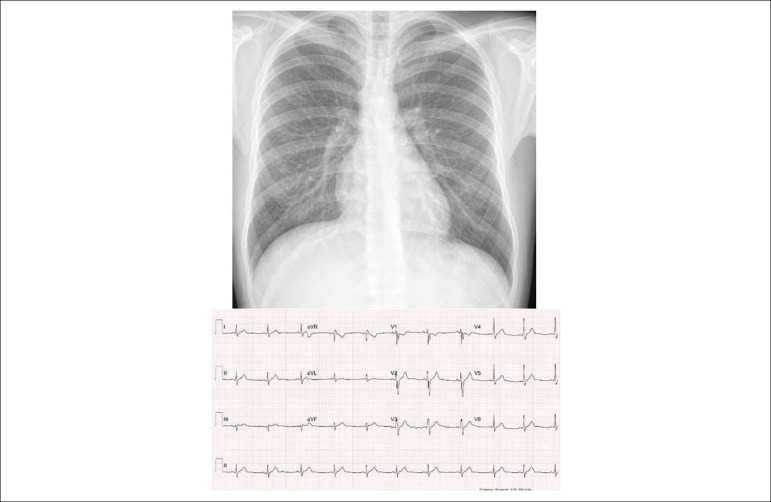
Chest X-ray emphasizes the cardiac area and the pulmonary vasculature within normal limits. The medial arch is rectified and electrocardiogram with signs of discrete final right bundle branch conduction disorder.

**Electrocardiography:** Sinus rhythm with right bundle branch conduction disorder, with polyphasic QRS complexes at V1 and thickened S waves from V4 to V6. Normal ventricular repolarization. AP =+ 60^o^, AQRS = -10^o^, AT+ + 60^o^. (Figure 1).

**Chest X-ray:** Normal cardiac area (CTI = 0.44) and normal pulmonary vasculature. The medial arch is rectified and the aortic knob slightly protruding (Figure 1).

**Figure 2:**
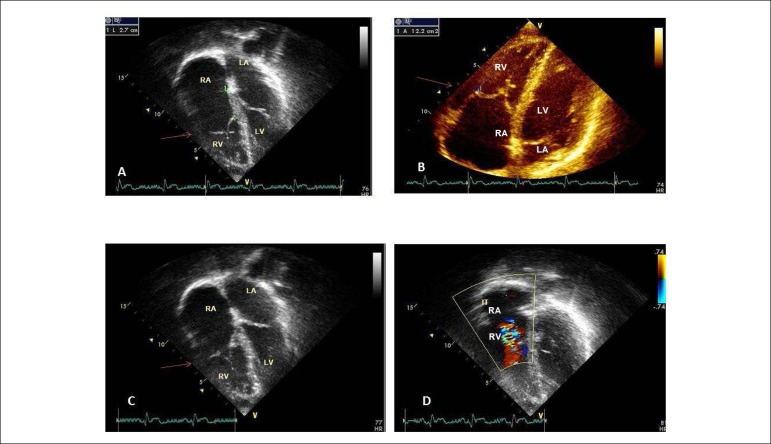
Echocardiogram shows, in the 4-chamber subcostal section, the septal and posterior valves sitting below the atrioventricular valvular plane, but with good coaptation in A, B, and C, and discrete tricuspid valve regurgitation in D. RA: right atrium; LA: left atrium; RV: right ventricle; LV: left ventricle.

**Echocardiography:** Normal atrioventricular connection with the tricuspid valve presenting apical implantation of its septal and posterior valves, inducing atrialization of a portion of the RV. There was a slight regurgitation of this valve and the heart cavities were normal in size. Aorta = 16, LA = 29, RV = 22, LV = 42, septum = posterior wall = 8 mm, LVEF = 60% (Figure 2).

**Nuclear magnetic resonance imaging:** Normal cardiac chambers. Tricuspid valve sitting low, 25 mm from the mitral annulus plane with atrialization of a portion of the RV cavity. RV diastolic volume was 53.5 ml. RV function was preserved (45%) as well as left ventricular function (73%). There was no late enhancement.

**Holter:** Supraventricular extrasystoles (3% of the total) and no supraventricular or ventricular tachycardias.

**Ergospirometry:** Maximum oxygen consumption of 40.1 ml/kg/min.

**Clinical diagnosis:** Ebstein’s anomaly with pronounced displaced of the septal and posterior valve but with minimal tricuspid valve regurgitation in an asymptomatic adult with previous ventricular ablation of anomalous right posterior pathway.

**Clinical reasoning:** There were no clinical elements of diagnostic orientation of Ebstein’s anomaly, given the absence of characteristic elements, mainly represented by tricuspid regurgitation. The displaced septal and posterior valves were well coupled to the extent of preventing regurgitation to the right atrium, hence the discrete repercussion of the congenital defect. The only retrospective diagnostic element was the anomalous right posterior pathway, which is common in Ebstein’s anomaly, which often causes supraventricular paroxysmal tachycardia. Diagnosis was well established by echocardiography and nuclear magnetic resonance imaging.

**Differential diagnosis:** Other heart diseases of discreet repercussion can also be presented this way. Hence the diagnostic difficulty in acyanogenic cardiopathies, such as atrial septal defect and persistent ductus arteriosus without heart murmurs, with complementary tests showing no abnormal findings. Interventricular septal defect usually shows a characteristic systolic murmur at the left sternal border, as well as obstructive defects such as pulmonary and aortic stenosis and aorta coarctation.

**Management:** As the clinical repercussion it is shown to be discreet, with no harm to ventricular function or blood disorders, with good balance of pulmonary and systemic flows over time, no signs of hypoxemia and/or heart failure and good physical tolerance, clinical expectant management was considered.

**Comments:** The natural evolution of this patient to adulthood demonstrates favorable elements in good clinical and hemodynamic conditions, except for the presence of anomalous bundles that could be eliminated by ablation. There were no acquired characters resulting from Ebstein’s anomaly, which is so common in this anomaly, from evolutionary time to adulthood. This is because this patient had no significant tricuspid regurgitation due to perfect fit of the septal and posterior tricuspid valves, although clearly sitting low in the RV cavity. Since deterioration has not been expected, expectant management is undoubtedly the most appreciated one.

Similarly rare cases have been reported, a 56-year-old atrial flutter reversed with drugs;^1^ a 36-year-old with paroxysmal tachycardia controlled with drugs;^2^ and a 87-year-old reversed after ablation of accessory pathways.^3^ A 62-year-old asymptomatic patient, despite anatomical disorders.^4^ Another one with long survival described in the literature, who decompensated with tricuspid regurgitation at 79 years of age.^5^

This unique rare case in view of good clinical evolution, despite the clear congenital anomaly, makes us think about the surgical approach adopted in similar cases and at earlier ages, which could progress in the same way.
